# Microbial levels on street foods and food preparation surfaces in Mangaung Metropolitan Municipality

**DOI:** 10.4102/hsag.v26i0.1407

**Published:** 2021-01-29

**Authors:** Malerato Moloi, Gaofetoge G. Lenetha, Ntsoaki J. Malebo

**Affiliations:** 1 Department of Life Sciences, Faculty of Health and Environmental Science, Central University of Technology, Free State, Bloemfontein, South Africa; 2 Centre for Innovation in Learning and Teaching (CILT), Faculty of Health Sciences, Central University of Technology, Free State, Bloemfontein, South Africa

**Keywords:** foodborne microorganisms, microbial contamination, street foods, surface swabs, food safety

## Abstract

**Background:**

The street food sector has become an important component of the food distribution system in many cities in both developing and industrialised countries, particularly for midday meals. However, certain street food can pose a significant risk to consumers because of microbiological contamination.

**Aim:**

The aim of this study was to determine the microbial levels of street foods and preparation surfaces in Mangaung Metropolitan Municipality.

**Setting:**

The study selected study setting was vending sites close to taxi ranks where prepared meals were accessible to consumers.

**Methods:**

The study was conducted in Mangaung Metropolitan Municipality during the winter season. Samples were collected through convenience sampling from the representative towns Thaba Nchu, Botshabelo and Bloemfontein. Using swabs, surface samples were collected and quantified from selective media. Eight beef samples were also collected; the microbial load on each sample was quantified and identified using a RapID kit.

**Results:**

The surface swabs obtained in Botshabelo (1.1 × 10^4^ cfu/m^2^ – 1.1 × 10^6^ cfu/m^2^) showed higher microbial counts as compared to those obtained in Bloemfontein (1.1 × 10^4^ cfu/m^2^ – 1.1 × 10^5^ cfu/m^2^) and Thaba Nchu (1.1 × 10^4^ cfu/m^2^ – 1.1 × 10^5^ cfu/m^2^). Higher microbial counts were observed on meat samples sampled in Thaba Nchu (50 cfu/g x 10^5^ cfu/g), Bloemfontein (48 cfu/g x 10^4^ cfu/g) and Botshabelo (33 cfu/g x 10^5^ cfu/g) when compared to international microbiological standards. After assessing the microbial levels, *Staphylococcus aureus, Escherichia coli, Candida guilliermondii, Corynebacterium jeikeium, Psychrobacter phenylpyruvicus* and *Peptostreptococcus tetradius* were identified.

**Conclusion:**

This study confirmed contamination of surfaces and food served by vendors in Mangaung Metropolitan Municipality. The identified foodborne bacteria could pose a public health problem in each specific locality.

## Introduction

According to the United Nations Food and Agricultural Organisation (FAO), about 2.5 billion people consume street food every day (Da Silva et al. 2014). Therefore, the demand to ensure the supply of safe food has been one of the major challenges and concerns for producers, consumers and public health officials globally (Da Silva et al. 2014). This is because foods excessively contaminated with pathogenic and spoilage microorganisms are undesirable and can cause foodborne illnesses (Hertanto et al. 2018). Transmission of pathogenic microorganisms to food takes place through poorly washed hands and dirty clothing, such as aprons that are worn during the preparation and serving of food (Hertanto et al. 2018). In addition, contaminated foods can endanger public health by causing various acute and chronic foodborne illness through pathogenic microbes or toxic substances present in them (Nazni & Jaganathan 2014).

Foodborne illness, also called ‘foodborne disease’, ‘foodborne infection’ or ‘food poisoning’, is a common, costly but preventable public health problem (Akter 2016). Foodborne illness can be caused by bacteria, parasites, toxins and viruses. Amongst the common pathogens are *Salmonella* and *Escherichia coli*, which account for 52 000 and 37 000 deaths, respectively, each year (WHO 2015). Each year, reports indicate that one in six Americans gets sick by consuming contaminated foods or beverages (Akter 2016). In South Africa, a progressive increase in listeriosis cases was noted starting in mid-June 2017, heralding what was to become the world’s largest listeriosis outbreak. A total of 1060 cases were reported for the period of 01 January 2017 – 17 July 2018 (Smith et al. 2019). Different disease-causing microbes, or pathogens, can contaminate foods. In addition, poisonous chemicals, or other harmful substances, can cause foodborne illness if they are present in food (Akter 2016).

The global burden of foodborne illness states that each year as many as 600 million, or almost 1 in 10 people in the world, fall ill after consuming food and water contaminated with viable pathogenic bacterial cells (or spores in the case of botulism) or food containing toxins produced by toxigenic bacteria and moulds (Akter 2016). Of these, 420 000 people die, including 125 000 children under the age of 5 years. Foodborne illnesses have devastating health implications, such as kidney and liver failure, brain and neural disorders, reactive arthritis and cancer, with fatal results (WHO 1996). Although acute gastrointestinal diseases are not all foodborne and foodborne illness does not always result in acute gastroenteritis, food does represent an important vehicle for pathogens causing acute gastroenteritis (Lamin-Boima 2017). Previous research done to assess the quality of street food in several countries has demonstrated that street food was the positive cause of foodborne illnesses (WHO 2010).

The statistics available in South Africa for the period 2001–2006 show that most epidemics were reported in the provinces of Eastern Cape, KwaZulu-Natal and Limpopo (DoH 2006). According to Shonhiwa et al. (2019), 327 foodborne illness outbreaks were reported from January 2013 to December 2017, causing illness in 11 155 individuals, with 78% hospital visits, 4% hospital admissions and 0.4% deaths. Most of the outbreaks were reported in the warmer months, from KwaZulu-Natal (43%), Gauteng (19%) and Mpumalanga (12%) provinces. Institutional outbreaks were most common (32%), followed by household outbreaks (27%) and community outbreaks (11%). However, Motarjemi (2013) believes that the majority of foodborne illness could be averted if food handlers were knowledgeable in safe food handling and if customers were informed correctly in their choices of food and food handling.

In Mangaung Metropolitan Municipality, many consumers buy street food daily, as it is both available and affordable. However, the microbial levels of these foods and their safety for human health are not well known. Knowing the bacteriological quality of street foods is an important factor in recognising the safety problems related to street foods (Amare et al. 2019). In the Mangaung metro area, street vendors are regulated by Mangaung Local Municipality by-laws relating to street trading as promulgated by Local Government Notice no. 4 of 20 January 2006 to ensure the safety of food. Therefore, this study seeks to investigate the microbial level of street food in the Mangaung metro area.

## Materials and methods

### Study area selection

Microbial samples were collected through non-probability convenience sampling in the Mangaung metro area in Free State, South Africa. The sampling method was chosen to conveniently select businesses that primarily sold cooked meat. Eight whole samples of cooked beef were randomly collected with dry and sterile polythene bags from different vendor stalls at different taxi ranks in Botshabelo, Thaba Nchu and Bloemfontein Central Business District. In addition, sterile surface swabs were used to collect samples from the preparation surfaces selected on the vending stalls, namely, food preparation areas and meat preparation areas. The samples were collected between 11:00 and 14:00, which is the usual holding period of the food. Afterwards, the samples were kept on ice during transportation to the laboratory. Then, the samples were placed in a refrigerator and analysed without delay. To assess factors that may contribute to the contamination of foods and surfaces, a checklist was used.

### Microbial quantification

For microbial quantification from meat samples, the beef samples were blended using a sterile blender, and then 10 g of beef samples were mixed with 90 mL of sterile peptone water and homogenised to make a 1:10 dilution. The surface swabs were also mixed with 90 mL of sterile peptone water and homogenised appropriately. Next, serial dilutions were done, the dilutions were prepared up to 10^6^ dilution, using a spiral plater. A spiral plater is an instrument used to dispense a liquid sample onto the Petri dish in a spiral pattern. The purpose of this method is to inoculate several dilution factors onto a single Petri dish. This allows a significant reduction in the number of dilutions performed and dishes used. About 1 mL of all dilutions was plated in triplicate Petri dishes using plate count agar (PCA). The plates were then incubated at 37 °C for 24 h. In addition, selective media were also used. Baird Parker was used for isolation of *Staphylococcus* species, mannitol egg yolk polymyxin (MYP) agar was used for isolation of *Bacillus* species and xylose lysine deoxycholate (XLD) agar was used for isolation of *Salmonella* species. After the appropriate incubation period, the plates were then examined for microbial growth (Sanders 2012).

### Microbial identification

The species of the foodborne pathogens were identified using a RapID identification kit (Thermo Fisher Scientific, SA). Briefly, organisms were streaked onto Petri dishes for isolation of pure cultures and used the next day. Afterwards, colonies were scraped from the agar plates using an inoculation loop into the RapID inoculation fluid to achieve the visual turbidity of a 2 McFarland standard. Next, the suspension was mixed thoroughly using the vortex. The contents of the suspension were then transferred to the RapID panel using a pipette. The test suspension was later mixed with the reaction cavities found in the RapID panel. Afterwards, the inoculated panels were incubated at 35 °C to 37 °C for 4 h. Following the incubation, two drops of RapID ONE reagent were added to cavities 15, 16 and 17. In additional, two drops of RapID spot indole reagent were added to Cavity 18. The cavities were then observed for colour development. The results of the colour development were recorded using the colour guide sheet and report form provided with the RapID kit. The results were recorded by scoring the colour development using either a plus or minus sign to indicate positive or negative reactions. The observed colour changes were a result of biochemical reactions. The numerical microbe code was then derived from the scores. These microbe codes were entered in the ERIC software for identification of the organism (Thermo Scientific SA).

### Ethical considerations

The study was approved by the Faculty Research and Innovation Committee. Ethical clearance was not needed in the current study as samples were collected from surfaced. Consent was requested from all vendors where samples were collected.

## Results and discussion

**FIGURE 1:**
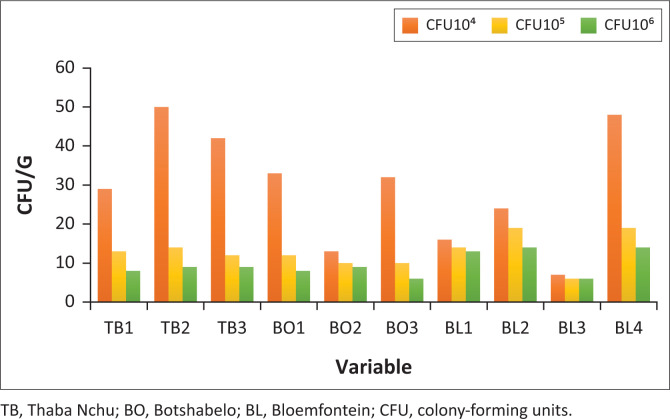
Total viable counts isolated from meat samples.

Based on the results found in the current study, Figure 1 represents the bacterial counts from meat samples collected from Mangaung metro area street vendors. The microbial levels of the meat samples grown on PCA medium are shown as dilutions from 1.1 × 10^4^ cfu/g to 1.1 × 10^6^ cfu/g. Bacterial counts obtained in Thaba Nchu (≤ 50 cfu/g × 10^5^ cfu/g), Bloemfontein (≤ 48 cfu/g × 10^5^ cfu/g) and Botshabelo (≤ 33 cfu/g × 10^5^ cfu/g). These results are higher when compared to the regulations governing microbiological standards for foodstuffs and related matters in South Africa, which stipulate that no person should sell meat for which the total colony count of organisms exceeds 10 000 per gram.

These high microbial counts are concerning, as they are an indication that proper food handling and preparation techniques are not always practised by food handlers. Common causes of food poisoning include failing to wash hands completely before preparing or eating food; using utensils, cutting boards or serving dishes that are not clean, causing cross-contamination; consuming dairy products or food containing mayonnaise that have been left out too long; and consuming foods that have not been stored or cooked at the right temperature, especially meat and poultry (Pietrangelo 2015). Infections caused by microorganisms are largely the result of the poor hygiene of the person responsible for preparing the food (Uçar, Yilmaz & Cakiroglu 2016). In addition, lack of good hygiene practices by food handlers has been reported to influence microbial rates and could also place consumers at risk of contracting foodborne illnesses, which may increase the statistical rates of deaths (Setlhare 2013). Okojie and Isah (2014) reported that street foods, when prepared in very dirty surroundings with wastewater and garbage disposed of nearby, provide nutrients and a breeding ground for rodents and other pests that could cause contamination of the food. Similar results were found in this study. During the survey, using a checklist to determine the status of the vending sites, it was observed from our results that most of these vending sites lacked basic infrastructure and services such as potable running water and waste disposal facilities; hand and dish washing water was usually insufficient and often reused. In most cases during the survey, running water was not available at vending sites; washing of hands and crockery was done in bowls or buckets and sometimes without soap. Furthermore, access to a frequent water supply was limited to facilities that had tap water. All these were considered violations of the regulations governing the general hygiene requirements for food premises and the transport of food and highlight a need for continuous monitoring of these businesses by environmental health professionals.

The regulations governing the general hygiene requirements for food premises and the transport of food (DOH 2002) stipulate that wash basins should always be provided, together with an adequate supply of soap and disposable paper towels. Therefore, street food vending stalls should have potable water available around the stall to make it easy for the vendors to access water. Note that most stalls are erected by vendors, with some erected by government in certain areas; this recommendation could work in areas where stalls have been erected by government or where stalls are erected close to business areas such as taxi ranks. The regulations also stipulate that no person should handle food intended for consumption if the hands of this person are not washed with soap and water. The lack of basic infrastructure to support the practice of hand washing was a matter of concern. This highlights a possible lack of compliance with proper hygiene practice and implies that the implementation of basic hygiene practices may be difficult because of the lack of hygiene infrastructure.

**FIGURE 2:**
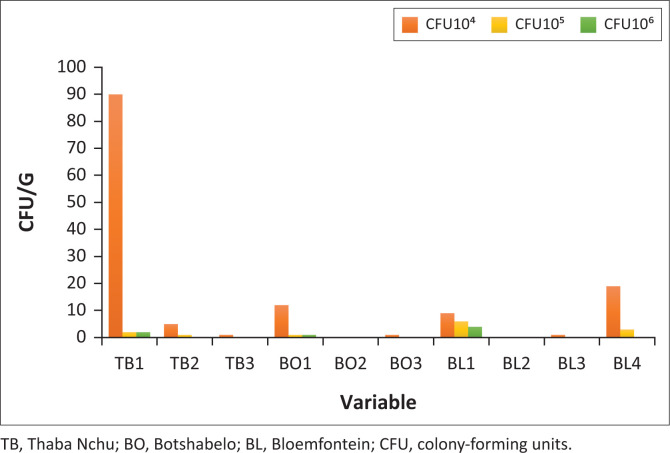
Total viable counts from meat samples for *Staphylococcus* isolates.

Microbial counts from meat samples at Bloemfontein, Thaba Nchu and Botshabelo (Figure 1) were found to be higher when compared to the regulations governing microbiological standards for foodstuffs and related matters in South Africa, which stipulate that no person should sell meat for which the total colony count of organisms exceeds 10 000 per gram. The results also showed the presence of *Staphylococcus* isolates (Figure 2), indicating that improper food handling practices, handling food with bare hands, reusing surfaces without cleaning first and not wearing aprons contributed to the presence of these foodborne pathogens. *S. aureus* is conveyed to food by the person handling it. Persons with skin, nose or throat infections or inflammatory wounds pass this microorganism onto the food (Uçar et al. 2016). The foods posing a particular risk for contamination with *Staphylococcus* spp. include cooked meat, potato salad, desserts with milk, such as custard, and chicken, fish and other meat salads (Duyff 2012). It is important to realise that food vendors can be carriers of pathogens such as *E. coli, S. aureus* and *Salmonella, Shigella* and *Campylobacter* spp. The food vendors eventually transfer these foodborne microbes to the consumers (Siddiqua 2016). Transfer can be direct, from person to person; indirectly in two stages, from person to contact surface and from contact surface to person; and indirectly from person to food and from food to person (Todd et al. 2007). Moreover, a lack of adequate infrastructure can affect the implementation of good hygiene practices.

**FIGURE 3:**
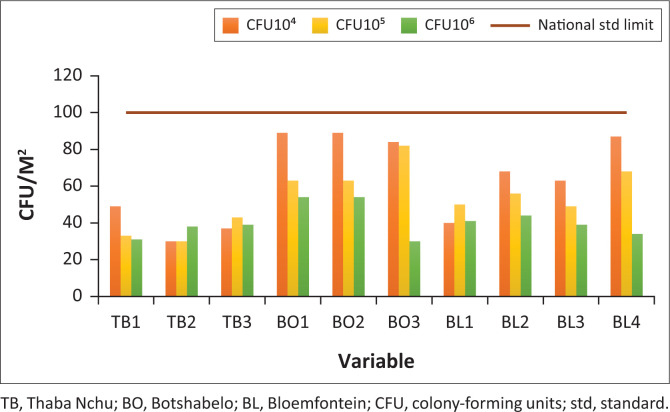
Total viable counts from preparation surfaces, collected using microbial swabs.

Figure 3 illustrates the total viable counts recorded on food preparation surfaces at various vending stalls in Thaba Nchu, Bloemfontein and Botshabelo. The samples obtained in Botshabelo (1.1 × 10^4^ cfu/m^2^ – 1.1 × 10^6^ cfu/m^2^) showed a higher microbial count compared to those of Bloemfontein (1.1 × 10^4^ – 1.1 × 10^5^ cfu/m^2^) and Thaba Nchu (1.1 × 10^4^ cfu/m^2^ – 1.1 × 10^5^ cfu/m^2^). This may be attributed to the fact that the vending stalls in Botshabelo are not next to paved roads, as opposed to those in Thaba Nchu and Bloemfontein. The vending stalls were not covered, and there was also dust from construction vehicles working not far from the stalls in Botshabelo. Even though the surface swab results from Botshabelo are a cause for concern, the counts were found to be lower than the South African national standard (100 cfu/m^2^).

After quantifying the microbial levels, the RapID identification method was used to identify foodborne pathogens that might be present in meat samples at the selected areas of Mangaung Metropolitan Municipality. The RapID results obtained (Table 1) indicate the presence of *Candida guilliermondii*, which is a yeast, and the bacteria *Corynebacterium jeikeium, Psychrobacter phenylpyruvicus* and *Peptostreptococcus tetradius*, amongst others. All these bacteria are found on human skin and might have been transferred from the hands of food handlers to the meat because none of the food handlers were seen wearing gloves during the food preparation and serving (Table 1). Such a food handler may have contaminated his or her hands when using the toilet and not washing hands, or bacteria might have spread from raw meat to green salads via the hands of the food handler (Kariuki 2018).

**TABLE 1:** Characterisation of foodborne pathogens isolated from meat samples.

Origin and sample no.	Species identified using RapID	Source	Health effects	Reference
Thaba Nchu 1	*Prevotella bivia*	Vaginal tract, oral flora	Endometritis and pelvic inflammatory disease	Mirza et al. 2012
Thaba Nchu 1	*Escherichia coli*	Undercooked or raw food, unpasteurised milk, apple juice, contaminated water	Bloodstream infection and urinary tract	CDC 2011
Thaba Nchu 1	*Psychrobacter phenylpyruvicus*	Human skin	Surgical wound infection	Deschaght et al. 2012
Thaba Nchu 2	*Corynebacterium jeikeium*	Human skin	Infection after disruption of the skin by surgery (cardiac or orthopaedic surgery)	Bechara et al. 2011
Botshabelo 1	*Escherichia hermannii*	Chickens and humans (blood, urine)	Bloody diarrhoea	Sedlock et al. 2018
Botshabelo 1	*Yersinia kristensenii*	Soil, freshwater, food	Human enteritis	-
Botshabelo 2 Botshabelo 3	*Peptostreptococcus tetradius*	Human skin	Clinical infection	Song et al. 2003
Bloemfontein 1 Bloemfontein 2	*Candida guilliermondii*	Human skin and mucosal surfaces	Chronic onychomycosis, septic arthritis and endocarditis	Girmenia et al. 2006
Thaba Nchu 3	*Staphylococcus aureus*	The exterior of a human ear and animals	Human skin infections, sialadenitis and food poisoning	Madigan & Martinko 2005
Thaba Nchu 2 Botshabelo 2	*Shigella* spp.	Salads (potato, tuna, chicken), raw vegetables, milk and dairy products, poultry	Blood or mucus in the stool, possible fever	Ghosh et al. 2007

*Staphylococcus aureus* was also identified on the meat samples of the three selected areas (Thaba Nchu, Botshabelo and Bloemfontein). The presence of *S. aureus* found in meat samples highlights the need for providing food handlers with educational training with respect to proper hygiene practices because humans are possible sources of the bacteria, as well as the need for provision of hand washing tools. Improper food hygiene practices are worrying, as *S. aureus* causes food poisoning with changes in blood pressure and pulse rate (Setlhare 2013). Staphylococcal disease results from eating food contaminated with toxins such as enterotoxin-producing strains of *S. aureus*, which leads to diarrhoea and vomiting. Staphylococci grow in food, in which they produce their toxins. Thus, staphylococcal food poisoning does not result from ingesting the bacteria but rather from ingesting the toxins that are already present in the contaminated food.

The results obtained (Table 1) also indicate the presence of *E. coli* in meat samples isolated from Thaba Nchu. This is a cause for concern because the presence of *E. coli* in food usually indicates recent faecal contamination (Kariuki 2018). Faecal coliforms appear in great quantities in the intestines and faeces of people and animals; hence, their presence in a food sample often indicates recent faecal contamination, meaning that there is a greater risk that pathogens may be present (Kariuki 2018). *Escherichia coli* has also been associated with contaminated water (CDC 2011). In addition, the water used by street vendors is not filtered; that is why it may contain bacteria and microorganisms such as *E. coli*. Moreover, *E. coli* might have been present in the contaminated water used by the food handlers, because they reuse water repeatedly, even when it is no longer clean to use it. This practice occurs because of the limited availability of hand washing infrastructure, which includes running water and basins for hand washing.

In addition to *E. coli, Shigella* spp. were isolated from the meat from both Thaba Nchu and Botshabelo (Table 1). According to the literature, *Shigella* spp. are frequently found in water polluted with human faeces (Dilbaghi & Sharma 2007). They can easily multiply at temperatures between 10 °C and 48 °C (Baş, Yüksel & Çavuşoğlu 2017). The optimum growth temperature for this bacterium is 37 °C (Warren, Yuk & Schneider 2007). Contamination of these foods usually takes place through the faecal–oral route. Faecally contaminated water and unsanitary handling by food handlers are the most common causes of contamination (Dilbaghi & Sharma 2007). In addition, improper waste disposal has been associated with the transmission of enteric pathogens like *Salmonella* spp., *Shigella* spp. and *E. coli*. Generally, vendors in informal settings do not have waste bins around the stalls; they use boxes and plastics to collect waste, and place these waste-collecting materials near the vending stalls. The presence of *Shigella* spp. might have resulted from the waste that was observed around the stalls. The most important protective factor against *Shigella* spp. is following proper personal hygiene rules (Uçar et al. 2016). Hand washing before handling food and thoroughly cooking all food before eating decrease the risk of getting shigellosis (Ram et al. 2008).

*Prevotella bivia*, which is found in the vaginal tract, was also isolated from the meat sample from Thaba Nchu. This finding is an indication that the food handlers do not wash their hands after using the toilet. They all (100%) indicated that the toilets were very far from their stalls, and that they must pay every time they use the toilets. These findings are consistent with a study conducted in Dhaka by Siddiqua (2016), where toilets were not available nearby in several cases, thus forcing vendors to eliminate their body wastes in nearby areas and return to their vending sites without washing their hands. In addition, in the current study, there was no potable water supply near the vending stalls; the food vendors obtained water from the nearby stores. The vendors stated that they only used the water for dish washing and cooking. During the survey, it was also observed that the vendors reused the dish washing water more than two times because they were trying to save the little water that they had.

Based on these results, Mangaung Metropolitan Municipality is urged to intervene and assist food handlers with the lack of basic infrastructure they are currently facing. This will be one of the keys to preventing cross-contamination and foodborne pathogens, which might lead to foodborne illnesses in the Mangaung metro area.

## Conclusion

The meat obtained in Thaba Nchu, Bloemfontein and Botshabelo showed high microbial counts. These results are alarming when compared to the regulations governing microbiological standards for foodstuffs and related matters in South Africa, which stipulate that no person should sell meat for which the total colony count of organisms exceeds 10 000 per gram. The results also showed the growth of *S. aureus*, indicating that the improper food handling practices carried out by food handlers contribute to the presence of these foodborne pathogens. The presence of pathogenic organisms such as *E. coli* is a cause for concern as it usually indicates recent faecal contamination. Therefore, consumption of such contaminated street vended foods poses a serious problem to community health.

In the current study, Table 1 shows that most of the bacteria identified are usually from the human skin and hair; the results may be an indication that the vendors are not practising food safety measures and hygiene, as transfer from the skin and hair can occur through touching surfaces and lack of hand washing when handling food. Consequently, vendors need to undergo food safety training to limit the spread of microbes through touching. Moreover, the lack of basic infrastructure (including nearby toilets) and services, such as potable running water and waste disposal facilities, as well as hand and dish washing water that is usually insufficient and often reused by food handlers, play an important role in the presence of foodborne pathogens identified in this study. Even though the surface swab counts were found to be lower than the South African national standard (100 cfu/m^2^), they are still cause for concern because they indicate that the food preparation surfaces are not being properly cleaned by food handlers

## Limitations

No swabs were taken of the food handlers’ hands.
